# Human iPS Cell-Derived Germ Cells: Current Status and Clinical Potential

**DOI:** 10.3390/jcm3041064

**Published:** 2014-10-13

**Authors:** Tetsuya Ishii

**Affiliations:** Office of Health and Safety, Hokkaido University, Sapporo 060-0808, Japan; E-Mail: tishii@general.hokudai.ac.jp; Tel.: +81-11-706-2126; Fax: +81-11-706-2295

**Keywords:** induced pluripotent stem cells, germ cells, primordial germ cells, oocyte, sperm, gametogenesis, meiosis, assisted reproductive technology, *in vitro* fertilization, intracytoplasmic sperm injection

## Abstract

Recently, fertile spermatozoa and oocytes were generated from mouse induced pluripotent (iPS) cells using a combined *in vitro* and *in vivo* induction system. With regard to germ cell induction from human iPS cells, progress has been made particularly in the male germline, demonstrating *in vitro* generation of haploid, round spermatids. Although iPS-derived germ cells are expected to be developed to yield a form of assisted reproductive technology (ART) that can address unmet reproductive needs, genetic and/or epigenetic instabilities abound in iPS cell generation and germ cell induction. In addition, there is still room to improve the induction protocol in the female germline. However, rapid advances in stem cell research are likely to make such obstacles surmountable, potentially translating induced germ cells into the clinical setting in the immediate future. This review examines the current status of the induction of germ cells from human iPS cells and discusses the clinical potential, as well as future directions.

## 1. Introduction

There are various reasons to generate germ cells from human pluripotent stem cells in the laboratory. First, *in vitro* recapitulation of gametogenesis and early embryogenesis using such induced germ cells is expected to enhance our understanding of the basis of human reproduction because the inaccessibility to human eggs (oocytes) and embryos has hampered relevant research. Second, human germ cell induction research will establish a precious platform for modeling infertility and congenital anomalies that have been difficult to study using animals. Third, the *in vitro* induction of germ cells from autologous pluripotent stem cells should lead to a new form of assisted reproductive technology (ART) for infertile patients who wish to have genetically-related children.

Recent advances in stem cell research have made it conceivable that human sperm (spermatozoon) and oocytes will be induced from pluripotent stem cells in the near future. Notably, a Japanese group reported that mouse embryonic stem (ES) cells and induced pluripotent (iPS) cells could be differentiated into fertile spermatozoa and oocytes via primordial germ cell (PGC)—like cells, and demonstrated that viable offspring could be derived from pluripotent stem cells [1,2]. Although their protocols used gonadal tissues and an *in vivo* induction system, their work established an important step on the path to the *in vitro* recapitulation of gametogenesis. Significant progress has also been made in the differentiation from both human ES cells [3,4,5,6,7,8] and iPS cells [8,9,10,11,12,13] into human germ cells over the last decade. A recent report demonstrated that human iPS cells can be indirectly or directly differentiated into the male germline, including haploid, round spermatid-like cells [10,12,13]. Rapid advances in stem cell research would help to overcome the current technical issues and lead to the *in vitro* formation of bona fide human spermatozoa and oocytes.

If functional oocytes and spermatozoa can be differentiated from human iPS cells, the use of such cells for research will contribute to the molecular elucidation of gametogenesis, as well as the onset and progression of various diseases in obstetrics, gynecology, and neonatology/pediatrics. However, with regard to the reproductive use of such germ cells induced from autologous iPS cells, sufficient preclinical research will need to be performed to confirm the safety of the offspring. Remarkably, the overview of ART (Appendix) using induced germ cells appears to occur against the Weismann barrier, wherein hereditary information moves only from germ cells to somatic cells [14]. Such germ cells are likely to be subject to genetic and/or epigenetic instabilities during iPS cell generation and germ cell induction. Moreover, although assessing the biological function of induced germ cells involves the creation of embryos and subsequent culture for a short period, human embryo research is strictly regulated in most countries [15]. In this review article, the current status of germ cell induction from human iPS cells is examined and discussed in light of clinical potential and future directions.

## 2. Clinical Implications of Germ Cell Induction *in Vitro*

Two fundamental cell types constitute multicellular eukaryotes. Somatic cells proliferate by mitosis and form the tissues and organs comprising the body. Germ cells undergo meiosis as well as mitosis, resulting in the generation of gametes that can transfer half the genetic material to the next generation. The lineage of germ cells is referred to as the germline.

If germ cells can be efficiently induced from human iPS cells, the availability of such germ cells could contribute to various biomedical fields. First of all, the research use of human female germ cells and embryos is largely difficult owing to ethical reasons and the scarcity of oocytes and embryos for research. In contrast, patient-specific induced germ cells can model diseases that are derived from aberrant germ cells or that occur during embryogenesis. A wide variety of somatic cells which are differentiated from patient-specific iPS cells have already been used for disease-modeling to enhance the understanding of the pathogenesis of diseases [16]. Currently, the low efficiency of the differentiation of human iPS cells into germ cells has hampered the unveiling of the molecular pathogenesis of various diseases, including germ cell tumors [17], aneuploidy, sex chromosome abnormalities [11], and female and male infertilities.

**Figure 1:**
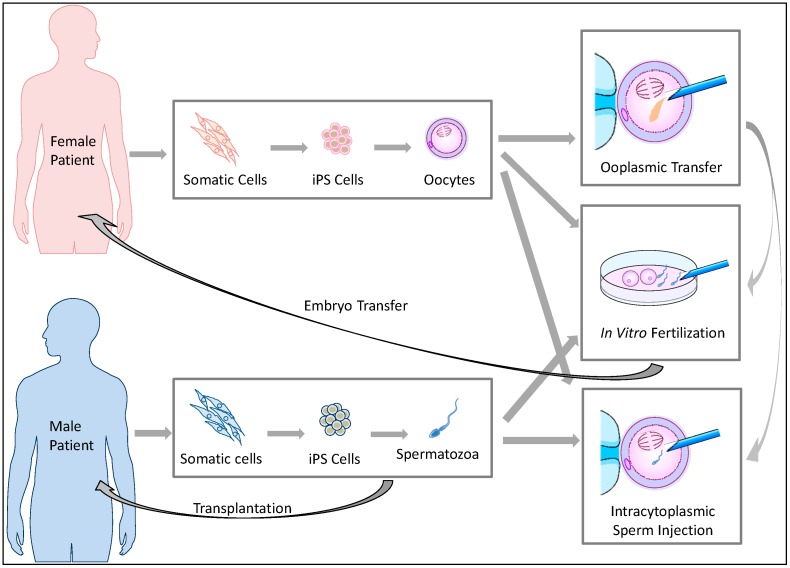
The potential reproductive uses of iPS cell-based germ cells. Autologous iPS cells can be generated from somatic cells biopsied from infertile patients who have lost viable oocytes or spermatozoa. Subsequently, germ cells are induced from the iPS cells. The regenerated germ cells can be used for *in vitro* fertilization or intracytoplasmic sperm injection to create embryos for transfer. In cases of male infertility, spermatogonial stem cells (SSCs) could be transplanted into patients to restore spermatogenesis potential. In cases of female infertility, ooplasmic transfer to enhance the viability of quality-compromised oocytes is conceivable if female germ cells with a sufficient number of mitochondria can be induced from iPS cells.

If functional germ cells are induced from iPS cells, such germ cells are also expected to impact ART treatment (Figure 1). Although ART has helped many infertile patients to produce offspring, the current ART procedures are based on the premise that an infertile couple can produce fertile gametes in order to perform intrauterine insemination (IUI), *in vitro* fertilization (IVF), or intracytoplasmic sperm injection (ICSI) (Appendix). Otherwise, the couple must use donor gametes. This option has raised ethical issues and social confusion. ART using donor gametes results in the birth of genetically-unrelated children. Such children born of donor gametes frequently confront stigma that stems from being uninformed about their genetic parents or due to their lack of resemblance to their parents in shape and appearance [18]. In addition, some sperm donors have anonymously provided their gametes to a tremendous number of patients, creating social problems [19]. Such cases frequently occur because there are many prospective parents who have no viable gametes due to congenital anomalies, or because they have been rendered sterile by receiving chemotherapy and radiation therapy for cancer treatment [20,21,22], or because the females have undergone age-related oocyte senescence [23].

Recent progress in germ cell induction research is increasing the possibility of a new form of ART using germ cells induced from autologous iPS cells for patients with no viable gametes (Figure 1). If fertile spermatozoa can be induced from a male patient’s iPS cells, performing IVF or ICSI will be possible using the generated spermatozoa. Similar approaches can be performed when fertile oocytes are generated from iPS cells. Even if no mature spermatozoa are obtained from the induction, *in vivo* spermatogenesis could be restored by transplanting spermatogonial stem cells (SSCs) derived from autologous iPS cells into the testis of a male patient [24,25,26]. In 1997, infusions of oocyte cytoplasm including mitochondria from donor oocytes was conducted in order to enhance the fertility of quality-compromised oocyte with mitochondrial defects [23], resulting in the birth of over 30 children [27]. However, the U.S. Food and Drug Administration concluded that further research was required for the use of this procedure in humans due to potential health risks to the progeny [28]. If this ooplasmic transfer procedure is sufficiently improved and induced female germ cells which genetically match the patient’s oocytes can be obtained from iPS cells, such germ cells could be used as a resource for ooplasmic transfer. Following such ART procedures, the resulting embryos can be carefully examined for three to five days post-conception, and one or more viable embryo(s) can then be selected for embryo transfer. Thus, autologous iPS-derived germ cells are expected to meet the reproductive needs of infertile couples who have lost viable gametes for medical reasons or aging but wish to have genetically-related children.

## 3. The Induction of Germ Cells from iPS Cells

**Figure 2:**
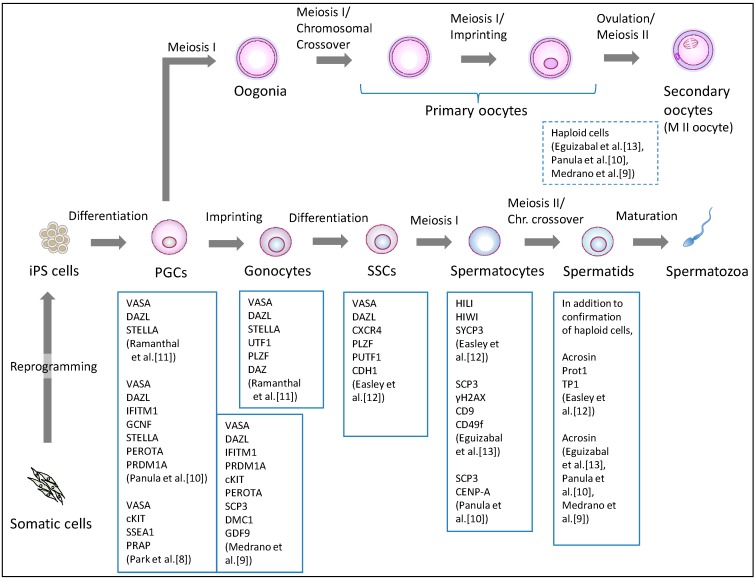
Differentiation pathway from human iPS cells to germ cells. Human iPS cells are differentiated into primordial germ cells (PGCs), and further differentiated into meiotic cells. Indicated information regarding confirmed markers is derived from research reports regarding germ cell induction using human iPS cells. PGCs: primordial germ cells, SSCs: spermatogonial stem cells.

Human iPS cells were initially generated from somatic cells by the ectopic expression of four transcription factor genes (OCT4, SOX2, KLF4, and c-MYC) in 2007 [29]. The current iPS cell generation methods vary in the choice of somatic origin, the set of reprogramming factors, and the transduction methodology [30]. The new pluripotent stem cells have become the starting material for germ cell induction, in which ES cells had been used (Table 1). Clinical applications of iPS-derived germ cells require scientific scrutiny in terms of meiosis, epigenetic programming, and the organization of the nucleus and mitochondria. Based on lessons learned from previous research on human ES cells [3,4,5,6,7,8] (Table 1), non-human primate ES cells [31], and mouse pluripotent stem cells [1,2,32,33,34,35], the current primary differentiation strategy involves differentiating human iPS cells into PGCs, and subsequently directing the PGCs to undergo meiosis, with some variations (Table 1). The PGC formation has been verified by the expression of marker genes or immunostaining for marker proteins including VASA (DDX4), cKIT, and SSEA1 (Figure 2). Confirming entrance into meiosis involves assessing the haploidy of differentiated cells as well as detecting meiosis-associated markers, such as acrosin, transition protein 1 (TP1), and protamine 1 (Prot1).

**Table 1:** Induction of germ cells from human pluripotent stem cells *in vitro* or *in vivo*.

Differentiation Method	Cell Lines Used			Differentiation Stage			Remarks	References
	iPS Cells	ES Cells		PGCs	Meiotic Cells	Haploid Cells		
EB formation	-	HSF-6(XX) HSF-1(XY) H9(XX)		-	-	-	Germ cell-like cells expressing VASA, SCP1, SCP3, BOULE, TEKT1, and GDF3 were observed.	Clark * et al.*, 2004 [3]
EB formation	-	NTU1(XX) NTU2(XX) NTU3(N.D.)		-	-	-	Germ cell-like cells expressing cKit, STELLA, VASA, and GDF9 were observed.	Chen * et al.*, 2006 [4]
Making colonies of fewer than 50 cells	-	HSF-6(XX) H9(XX)		Yes	-	-	Sertoli-like cells were simultaneously generated in this process.	Bucay * et al.*, 2008 [7]
Monolayer differentiation and FACS enrichment of SSEA1-positive cells	-	H9(XX) hES-NCL1(XX)		Yes	-	-	PGCs with removal of parental imprints and chromatin modification changes were generated.	Tilgner * et al.*, 2008 [6]
Differentiation on primary human fetal gonadal stromal cells, and isolation of a triple biomarker (cKIT, SSEA1, VASA)—positive cells	hIPS2(XY) hIPS1(XY)	HSF-6(XX) HSF-1(XY) H9(XX)		Yes	-	-	PGCs derived from human iPS cells did not initiate imprint erasure as efficiently.	Park * et al.*, 2009 [8]
Overexpression of DAZL, DAZ and BOULE following induction by BMPs	-	HSF-1(XY) HSF-6(XX) H1(XY) H9(XX)		Yes	Yes	Yes	DAZL functions in PGC formation, whereas DAZ and BOULE promote later stages of meiosis and development of haploid gametes.	Kee * et al.*, 2010 [5]
Overexpression of DAZ, DAZL, and BOULE following induction by BMPs	iPS(IMR90) (XX) iHUF4(XY)	H9(XX) HSF-1(XY)		Yes	Yes	Yes	Fetal-derived iPS cell line iPS (IMR90) and adult-derived iPS cell line iHUF4 were generated by lentiviral transfection with OCT3/4, SOX2, KLF4 and c-MYC.	Panula * et al.*, 2011 [10]
Overexpression of VASA and/or DAZL following differentiation on matrigel-coated plates	iPS(IMR90)(XX) iHUF4(XY)		iHUF3(XX) iHUF4(XY)	Yes	Yes	Yes	The same iPS cell lines described in Panula * et al.* were used.	Medrano * et al.*, 2011 [9]
Two step protocol: Culture in bFGF-depleted ES cell media, subsequently, RA added; Sorted cells are cultured with FRSK, rLIF, bFGF, and R115866	KiPS1(XY) KiPS2(XY) KiPS3(XY) KiPS4(XX) CBiPS1(XY) CBiPS2(XY) CBiPS3(XX) CBiPS4(XY) CBiPS5(XX)		HS306(XX) ES[6](XY)	-	Yes	Yes	iPS cells of different origin (keratinocytes and cord blood) were generated with a different number (2–4) of transcription factors.	Eguizabal * et al.*, 2011 [13]
Direct differentiation using mouse SSC culture conditions	H1(XY)		HFF1(XY)	-	Yes	Yes	iPS cells derived from male foreskin fibroblasts were used.	Easley * et al.*, 2012 [12]
1. Differentiation into PGCs with BMPs, RA, and hrLIF. 2. Induction of gonocytes by transplanting iPS cells directly into murine seminiferous tubules *in vivo*	iAZF1(XY) iAZF2(XY) iAZFΔbc(XY) iAZFΔc(XY) iAZFΔa(XY)		H1(XY)	Yes	-	-	iPS cells derived from dermal fibroblasts of males with intact Y chromosome (iAZF) and Y chromosome deletions (iAZFΔ) were used. Gonocytes expressing VASA, STELLA, UTF1, PLZF, and DAZ were induced.	Ramanthal *et al.*, 2014 [11]

PGCs: primordial germ cells; EB: embryoid body; BMPs: bone morphogenetic proteins; bFGF: basic fibroblast growth factor; FRSK: Forskolin; RA: retinoic acid; hrLIF: human recombinant leukemia inhibitory factor; SSC: spermatogonial stem cell.

### 3.1. Induction of the Male Germline

The differentiation of human male iPS cells has so far produced PGCs [8,9,10], gonocytes [11], SSCs [12], spermatocytes [12,13], and haploid, round spermatid-like cells [10,12,13] (Figure 2, Table 1). As early as 2009, Park *et al.* [8] reported PGC induction from human iPS and ES cells. They used a triple biomarker (cKIT, SSEA1, VASA) assay to identify and isolate the PGCs, and demonstrated that culturing such human pluripotent stem cells on human fetal gonadal stromal cells, which were derived from a 10-week-old human fetus, significantly improved the efficiency of PGC formation. Moreover, the efficiency was comparable among various ES cell and iPS cell lines. Utilizing bisulfite sequencing, they showed that the PGCs initiate imprint erasure from differentially methylated imprinted regions (H19, PEG1, and SNRPN DMRs) by day seven of differentiation. However, PGCs derived from iPS cells did not initiate imprint erasure as efficiently, suggesting that further investigation is needed on the epigenetic status during germ cell induction from iPS cells.

In 2011, Panula *et al.* compared the potential of human iPS cells, derived from adult and fetal somatic cells to form primordial and meiotic germ cells [10]. As a consequence, approximately 5% of human iPS cells were found to have differentiated into PGCs with induction by bone morphogenetic proteins (BMPs). In addition, by overexpressing intrinsic regulator genes, including *DAZ*, *DAZL*, and *BOULE*, iPS cells formed meiotic cells with extensive synaptonemal complexes and post-meiotic haploid spermatid-like cells. These results show that human iPS cells generated from adult somatic cells can form germline cells other than PGCs. More recently, similar results using the overexpression of *VASA* and/or *DAZL* was reported, demonstrating that both human ES cells and iPS cells differentiated into PGCs, and the maturation and progression of these cells through meiosis was enhanced [9]. Again, post-meiotic male haploid cells were induced in 14 days following the overexpression of the two regulators. Moreover, the methylation pattern of the H19 locus, similar to that of normal germ cells, was observed following the expression of *VASA* alone. Therefore, such RNA-binding proteins appear to promote the meiotic progression of human iPS cell-derived germ cells *in vitro*.

In contrast to these studies, Eguizabal *et al.* demonstrated that without the overexpression of germline related factors, postmeiotic haploid cells were consistently obtained from human iPS cells of different origins (keratinocytes and cord blood), generated with a different number of transcription factors [13]. Their two-step differentiation protocol begins with iPS cell culture for three weeks with human ES cell media in the absence of bFGF. Subsequently, retinoic acid (RA) is added to the medium, and the culture continues for three more weeks. Then, the cells are sorted and reseeded onto culture plates in the presence of forskolin (FRSK), human recombinant leukemia inhibiting factor (rLIF), bFGF, and the CYP26 inhibitor, R115866, for at least two weeks. Consequently, the post-meiotic spermatid-like cells with acrosin-staining were identified. Moreover, Easley *et al.* also reported a similar direct differentiation approach without the overexpression of genes [12]. They adopted standardized mouse SSC culture conditions [36] and demonstrated that human ES cells and iPS cells differentiated directly into advanced male germ cell lineages, without genetic manipulation. They observed spermatogenesis *in vivo* by differentiating these pluripotent stem cells into UTF1-, PLZF-, and CDH1-positive spermatogonia-like cells; HIWI- and HILI positive spermatocyte-like cells; and haploid, round spermatid-like cells expressing acrosin, TP1, and Prot1. Such spermatids had uniparental genomic imprints similar to those of human sperm on two loci: H19 and IGF2. These results demonstrate that male iPS cells have the ability to differentiate directly into haploid, round spermatids *in vitro*.

Therefore, male germ cell induction from iPS cells has rapidly advanced since 2009. Although transplantation of autologous SSCs to restore spermatogenesis has already succeeded in infertile monkeys [37], clinical use of SSCs induced from iPS cells requires considerable caution. Notably, Amariglio *et al.* warned that transplantation of stem cells, not differentiated cells, in a patient could cause an adverse event [38]. They reported that a boy with ataxia telangiectasia treated with the intracerebellar and intrathecal injection of human fetal neural stem cells was diagnosed with a multifocal brain tumor four years after the first injection. One might consider using iPS cell-derived spermatids in the clinical setting. However, although oocytes have been fertilized with elongated spermatids [39,40], they were insufficiently fertilized with premature, round spermatids, resulting in poor embryonic development [41,42,43]. Successful fertilization of oocytes with more matured male germ cells *in vitro* needs to be examined in preclinical research.

Based on the recent mouse work by Hayashi *et al.* [1], primordial germ cell-like cells (PGCLCs) were generated from ES cells and iPS cells through epiblast-like cells (EpiLCs), a cellular state highly similar to pregastrulating epiblasts but distinct from epiblast stem cells (EpiSCs). To examine whether such PGCLCs undergo proper spermatogenesis, PGCLCs were transplanted into the seminiferous tubules neonatal mice lacking endogenous germ cells. As a result, fertile spermatozoa were produced in the thick tubules. The global transcription profiles, epigenetic reprogramming, such as imprinted genes (*Igf2r*, *Snrpn*, *H19*, and *Kcnq1ot1*), and cellular dynamics during PGCLC induction from EpiLCs resembled those associated with PGC specification from the epiblasts. Remarkably, they identified Integrin-b3 and SSEA1 as markers that could be used to isolate PGCLCs from differentiated cells. More recently, Ramathal *et al.* demonstrated that human iPS cells transplanted directly into mouse seminiferous tubules differentiated extensively to form germ cell-like cells with morphology indistinguishable from that of fetal germ cells, and these cells expressed PGC-specific proteins including VASA, DAZL, and STELLA [11].

These findings revealed the significance of differentiation pathway from iPS cells to germ cells and elaborated the need for culture conditions that mimic the stem cell niche in the testis to efficiently and effectively direct human iPS cells to form more advanced germ cells *in vitro*.

### 3.2. The Induction of Female Germline

In contrast to male germline induction, the differentiation of iPS cells or ES cells into female germ cells has been insufficiently studied (Table 1, Figure 2). Eguizabal *et al.* consistently observed between 1.0%–2.0% haploid cells per human female iPS cell line (derived from keratinocytes or cord blood) in their two-step differentiation protocol [13]. Their female iPS cells were differentiated into haploid cells following the detection of the SCP3 and H2AX proteins (indicators of meiotic competence). However, they observed that most of the iPS cell lines, including female cells, increased their methylation status of H19 (the maternally expressed, paternally imprinted gene), displaying a clear tendency toward paternal imprinting. Therefore, it appears that the germ cells induced from female iPS cells are certainly haploid, but are incomplete as mature female germ cells because oocytes only extrude the last polar body after fertilization. Panula *et al.* also reported a similar result regarding the differentiation of female iPS and ES cells into meiotic germ cells by the overexpression of the intrinsic regulators [10]. Moreover, Bucay *et al.* showed that germ cells differentiated from human ES cells *in vitro* express both male and female genetic programs regardless of their karyotype [7].

With regard to mouse systems, there have been attempts to induce female germ cells from ES cells since 2003 [44,45,46,47,48]. Although a follicle-like structure with oocyte-like cells was spontaneously observed, entrance into meiosis was not confirmed in those reports. Nicholas *et al.* clearly noted that mouse ES cell-derived oocyte maturation ultimately fails *in vitro* [48]. They transplanted ES cell-derived oocyte-like cells into an ovarian niche to direct their functional maturation and showed that the physiological niche of the ovary is required for their differentiation. Notably, Hayashi *et al.* showed that mouse female ES cells and iPS cells were differentiated into fertile oocytes via EpiLCs and PGCLCs [2], using a combined *in vitro* and *in vivo* system which led to the successful induction of fertile spermatozoa in 2011 [1]. When the PGCLCs were aggregated with female gonadal somatic cells as reconstituted ovaries, they underwent X-reactivation, imprint erasure, and cyst formation, and exhibited meiotic potential. After PGCLCs in the reconstituted ovaries were transplanted under the mouse ovarian bursa, such cells matured into germinal vesicle-stage oocytes, and contributed to fertile offspring after *in vitro* maturation and fertilization. Therefore, the differentiation of female iPS cells into germ cells largely depends on the use of an *in vivo* system.

The difficulty in female germ cell induction from pluripotent stem cells is more likely to reflect *in vivo* oogenesis. Human gametogenesis initiates around the 23–26th day post-conception [49]. The precursors of gametes, the PGCs, appear in the dorsal wall of the yolk sac near the developing allantois. The PGCs proliferate and migrate through the dorsal mesentery into the gonadal ridges. The PGCs are found in the gonads by the fourth week post-conception. Thereafter, female and male PGCs differentiate into oogonia (subsequently, oocytes) or gonocytes (subsequently, spermatozoa), respectively. The male germ cells undergo mitotic arrest until birth, whereas the female germ cells further enter meiotic arrest (Figure 2). Following birth, such germ cells are reactivated and resume meiosis, resulting in the beginning of the production of mature oocytes and spermatozoa after puberty. Therefore, human gametogenesis proceeds on a long-term basis with gender differences in meiotic progression.

In males, SSCs are maintained, and contribute to spermatogenesis by self-renewal *in vivo* for a long time. In addition, human SSCs can be maintained *in vitro* for a long term. Sadri-Ardekani *et al.* demonstrated that the human SSC numbers increased 53-fold within 19 days in testicular cell culture and increased 18,450-fold within 64 days in a germline stem cell subculture [50]. Conversely, it has generally been considered that most female germ cells enter meiosis I until birth, do not proliferate after birth, and that the number of the germ cells gradually declines until menopause (at approximately 40 years) [51]. The significant differences in the proceedings between spermatogenesis and oogenesis appear to impact the differentiation of human iPS cells into germ cells in the laboratory. However, there have been several unique reports regarding mammalian oogenesis. Some groups have reported the isolation of oogonial stem cell-like cells in mice and humans [52,53,54,55]. However, there are counterarguments about the existence of oogonial stem cells [56,57,58]. If the mitotically active oogonial cells can be isolated in a reproducible manner, the findings are expected to contribute to enhancing female germ cell induction as well as providing a mitochondrial resource for ooplasmic transfer.

## 4. Future Directions

In order to improve the induction efficiency and functional completeness of germ cell induction from human iPS cells, deeper insight into iPS cell generation and gametogenesis *in vivo* is vital. In addition, creating human embryos is likely to require the assessment of the developmental potential of induced germ cells. The conditions to permit the creation of human embryos for these functional assays should be discussed, because such experiments are frequently associated with ethical concerns or issues [15].

### 4.1. Genetic and Epigenetic Stability of Human iPS Cells

As mentioned in the Introduction, the ART using induced germ cells appears to be against the Weismann barrier. Induced germ cells are likely to be subject to genetic and/or epigenetic instabilities during iPS cell generation and germ cell induction. The genetic stability of iPS cells significantly impacts their research use, in addition to their safe medical use. Some cytogenetic analyses have suggested that human iPS cells and ES cells are likely to acquire trisomies in chromosome 12, and 17, indicating an underlying mechanism of growth advantage associated with culture adaptation [59,60,61]. Moreover, the tendency for large-scale chromosomal aberrations appears to have no dependence on the cell origin or iPS generation methods, although some of the chromosomal aberrations observed in PS cells were derived from the original somatic cells [59,60,62]. In addition, human iPS cell cultures are likely to undergo chromosomal changes at both early and late passages. A close examination of the genetic changes during culture indicated that the observed peak in occurrence of chromosomal aberrations is at around passage eight in iPS cells, while that in ES cells is at around passage 36 [59]. Moreover, smaller copy number variations (CNVs) in human iPS cell culture are present across chromosome 12, 17, and 20 [63]. Compared with human ES cells, iPS cells showed increased CNVs, and had more CNVs at low passages (18%) than at late passages (9%) [62,64]. Therefore, human iPS cells seem to be subject to genetic changes at earlier culture stage, mostly resulting from somatic cell reprogramming.

The genetic instabilities might occur not only in nuclear DNA, but also in mitochondrial DNA (mtDNA). The copy number of mtDNA, which encodes proteins required to produce ATP for motility of spermatozoon ranges from 2.8 to 226 copies of mtDNA [65]. In contrast, in oocytes, the mtDNA copy number ranges from 20,000 to 598,000 [66,67,68,69], significantly impacting the outcome of fertility in ART. Since proper ATP production by mitochondria is essential for accurate meiosis in oogenesis as well as normal embryonic development [66,67,68,69], the mtDNA integrity of human iPS cells needs to be addressed. Relative to the founder fibroblasts, a higher rate of heteroplasmic variation was observed in human iPS cells [70]. Although this phenomenon may imply an increased mutation load in the iPS cells, such iPS cell lines showed no significant metabolic differences. Van Haute *et al.* tested 16 human ES cell lines and showed that they carry a plethora of diverse mtDNA deletions [71]. The mtDNA mutations did not seem to correlate with the time in culture, and were detected in the early passage cells. Such deletions did not appear to impact the differentiation potential, and were still present in terminally differentiated cells. Conversely, Wahlestedt *et al.* reported a unique result using a mutator mouse model with an error-prone mtDNA polymerase [72]. They investigated the impact of an established mtDNA mutational load regarding the differentiation properties of mouse iPS cells. As a consequence, the mutator iPS cells displayed delayed proliferation kinetics and harbored extensive differentiation defects, although somatic cells with a heavy mtDNA mutation burden were amenable to reprogramming into iPS cells. These findings suggest the need for careful analyses of the nuclear DNA and mtDNA in human iPS cells prior to germ cell induction.

In addition, epigenetic aberrations in human iPS cells have been pointed out, indicating defects in DNA methylation, including regions subject to imprinting [73]. Interestingly, high-resolution DNA methylation profiles suggested that some iPS cell lines possess somatic memory [74,75]. Although iPS cell lines with such memory might readily differentiate into germ cells, careful assessment of the epigenetic status of human iPS cells is required to avoid a low efficiency differentiation or aberrant epigenetics in the resulting germ cells.

### 4.2. The Pluripotency State of Human iPS Cells

Human ES and iPS cells are more similar to mouse EpiSCs that were derived from epiblasts in postimplantation embryos than mouse naive, ground state ES cells [76,77]. The features of the ground state pluripotency include driving *Oct4* (also known as *Pou5f1*) transcription by its distal enhancer, globally reduced DNA methylation, prominent deposition of the repressive histone modification H3K27me3, and bivalent domain acquisition on lineage regulatory genes [78]. Moreover, human female ES and iPS cells frequently show a pronounced tendency for X chromosome inactivation. These lines of evidence suggest that human iPS cells represent a primed state of pluripotency that is distinct from the naive pluripotent ground state of mouse ES and iPS cells. Recently, some new methods to establish human iPS cells have been proposed [79,80,81]. These methods, which are based on 2i/LIF conditions (exogenous stimulation with leukemia inhibitory factor and small molecule inhibition of ERK1/ERK2 and GSK3β signaling) with additional components, demonstrated the establishment of human iPS cells in the naive ground state [79,81], or in the preimplantation epiblast state [80]. The use of human iPS cells generated by such methods are likely to facilitate the subsequent appropriate differentiation pathway to germ cells, as demonstrated by the two mouse experiments in which mouse pluripotent stem cells were differentiated into germ cells via EpiLCs [1,2].

### 4.3. Spatio-Temporal Factors in Gametogenesis

Currently, germ cell induction from human iPS cells is advancing primarily in the male germline. A better understanding of gametogenesis would facilitate the induction of female germ cells, as well as the terminal differentiation into spermatozoa. Following puberty, spermatogenesis occurs at the seminiferous tubules in the testis in which Sertoli cells co-exist with Leydig cells. In inducing male germ cells, co-culture with Sertoli cells that foster and differentiate spermatocytes *in vivo* has already been introduced to induce spermatogenesis *in vitro*. Park *et al.* improved PGC generation using a co-culture system with human fetal gonadal cells [8]. Moreover, Bucay *et al.* reported that PGC generation from human ES cells was accompanied by the development of Sertoli-like support cells [7]. Moreover, another article reported that testosterone, which the Leydig cells of the testes produce, was added to the culture medium in order to promote differentiation of mouse iPS cells into male germ cells *in vitro* [82]. More elaborate culture systems including Sertoli cells and Leydig cells may be effective to induce terminally differentiated male germ cells. However, fetal and adult populations of Leydig cells are distinct cells in terms of their physiology and function [83]. A recent report suggested that Sertoli cells support adult Leydig cell development in the prepubertal testis [84]. Regarding female germ cell development, oocytes are surrounded by a single layer of flattened ovarian follicular epithelial cells at meiotic arrest. When stimulated at puberty, the oocyte enlarges, and the follicular cells continue to proliferate to form many layers surrounding the oocyte. These cells eventually become known as granulosa cells that secrete progesterone after ovulation. Qing *et al.* have used co-culture with ovarian granulosa cells in the induction of oocyte-like cells expressing oocyte-specific genes including Figalpha, GDF-9, and ZP1-3 from mouse ES cells [47]. Interestingly, when they were co-cultured with Chinese hamster ovary (CHO) cells or cultured in CHO cell-conditioned medium, these cells did not express all of these oocyte-specific markers during the germ cell induction. Moreover, Nicholas *et al.* differentiated mouse Oct4-GFP ES cells *in vitro*, isolated GFP positive germ cells by FACS and co-aggregated the cells with dissociated mouse newborn ovarian tissue [48]. Subsequently, they transplanted the co-aggregates under the kidney capsule of recipient mice. They observed ES cell-derived Oct4-GFP positive oocytes in the graft despite the efficiency being low. Furthermore, in a recent work [2], Hayashi *et al.* differentiated PGCLCs, which were induced from mouse ES and iPS cells into fertile oocytes, by using *in vitro* aggregation with female gonadal somatic cells and transplantation of germ cells under the mouse ovarian bursa. The spatio-temporal factors associated with human gametogenesis *in vivo* should be further considered to develop more elaborate culture or differentiation systems in order to increase the possibility of inducing more mature germ cells from human iPS cells.

### 4.4. Assessing the Developmental Potential of Induced Germ Cells

In order to confirm whether induced human germ cells possess the correct biological functions, creating embryos and culturing them for a short term is indispensable prior to considering the use for clinical applications. In doing so, a subsequent biological analysis would necessitate the establishment of ES cells from the embryos. Nonetheless, these experiments are likely to raise ethical concerns owing to the fact that such embryos are created and destroyed for research purposes, not for reproduction. In some countries, creating a human embryo and monitoring the development of human embryos until the 14th day post-conception or until the beginning of the formation of the primitive streak may be permitted with approval of an institutional review board (IRB) and/or national authorities [15]. However, such human embryo experiments require sufficient data to support their use based on animal experiments to confirm scientific or medical rationality. Since non-human primate (NHP) experiments are more scientifically comparable with the human conditions than experiments in lower animals such as rodents, the data obtained from NHP experiments are likely to be required by IRB or other bodies with regard to granting permission for human embryo research.

## 5. Conclusions

As discussed above, human germ cell induction has advanced primarily in the male germline, progressively reaching to a final differentiation stage. Meticulously selecting human iPS cell lines with higher pluripotency and genetic integrity is expected to improve the efficiency of the formation of PGCs and entrance into meiosis. Moreover, placing the differentiated cells in culture systems similar to the niche in human gonadal tissues will likely produce not only spermatozoa, but also female germ cells that are more similar to oocytes. Further considerations of the intrinsic regulators that could be overexpressed are also likely to advance meiotic progression, complete meiosis, and functionally mature these germ cells.

Rapid advances in stem cell research will likely enable human iPS cells to differentiate into elongated spermatids or *bona fide* spermatozoa within the next decade or less. Recently, perplexing ethical and social concerns associated with the careless use of induced germ cells have been raised [15]. The use of ART with induced germ cells might facilitate posthumous conception, the birth of many siblings in a region without their knowing their genetic relationships, and facilitating the birth of a “savior sibling” to provide HLA-matched transplantation therapy for a relative. The uncontrolled or unethical use of induced germ cells would make the current problems associated with ART more complicated. As human germ cell induction from human iPS cells proceeds, appropriate deployment of this stem cell technology in ART will become an urgent matter that will need to be addressed by both researchers and the general public, including prospective parents.

## Appendix

### Assisted Reproductive Technology (ART)

ART involves several types of medical procedures to achieve pregnancy. Types of ART include IUI, oocyte retrieval, IVF, and ICSI.

### Intrauterine Insemination (IUI)

At the early stage of ART, IUI is performed by placing spermatozoa inside a woman’s uterus in order to facilitate fertilization.

### *In Vitro* Fertilization (IVF)

IVF begins with the induction of ovulation via hormonal stimulation, followed by oocyte retrieval. Subsequently, the retrieved oocytes are fertilized with spermatozoa in a petri dish. The resulting embryos are cultured for three to five days following fertilization, and one or more viable embryo is transferred to the uterus.

### Intracytoplasmic Sperm Injection (ICSI)

In cases of male infertility, one spermatozoon is generally injected into an oocyte to facilitate fertilization under a microscope. The embryos are cultured and transferred as in IVF.

### Ooplasmic Transfer [23,27,28]

In cases of female infertility, ooplasm, including mitochondria from fresh, mature or immature, or cryopreserved-thawed donor oocytes are directly injected into recipient oocytes via a modified ICSI technique to enhance the viability of the oocytes. Currently, there is a moratorium on this procedure in the U.S. and Canada due to the potential health risks to the progeny.
